# Does Resilience Correlate With Mental Health Following Flexor Hallucis Longus Tendon Transfer for Achilles Pathologies?

**DOI:** 10.7759/cureus.98895

**Published:** 2025-12-10

**Authors:** Simon Lalehzarian, LaMiah Hall, Tyler Kelly, Garrett Jebeles, Marc Bernstein, Keerthi Jaliparthy, Aarvi Shah, Apurv Gabrani, Fabio Pencle, Ashish Shah

**Affiliations:** 1 Department of Orthopedic Surgery, University of Alabama at Birmingham, Birmingham, USA

**Keywords:** brief resilience scale, fhl transfer, flexor hallucis longus transfer, mental health, patient-reported outcomes, promis, resilience

## Abstract

Background: Resilience is a person’s ability to recover from a perceived stressor. Our paper aims to describe trends in resilience and to describe the relationship between resilience and mental health following flexor hallucis longus (FHL) tendon transfer for Achilles pathologies.

Methods: Brief Resilience Scale (BRS) and Patient-Reported Outcomes Measurement Information System Mental Health (PROMIS MH) scores were obtained prospectively and analyzed retrospectively. The Foot and Ankle Ability Measure (FAAM) was also assessed to provide a reference for postoperative changes in foot-related physical function during activities of daily living (ADL) and sports. Descriptive statistics were used to assess demographics as well as changes in resilience and PROMIS MH.

Results: Preoperative scores for BRS and PROMIS MH were 3.65 (0.8046) and 49.22 (9.37). Resilience showed an increase at the two-week follow-up to 3.66 (p=0.6692). PROMIS MH significantly increased at the two-week interval from 49.22 to 50.11 (p=0.0001). Resilience was found to be significantly associated with PROMIS MH (p < 0.05). Despite gradual improvements in FAAM-ADL and FAAM-Sports, postoperative PROMIS MH scores did not indicate a significant improvement in mental health beyond baseline up to one year postoperatively.

Conclusions: Resilience shows no changes in value between the preoperative and postoperative state for FHL transfer and displays a significant correlation with PROMIS MH.

## Introduction

Flexor hallucis longus (FHL) tendon transfer was first described in 1991 and has gained popularity for treating Achilles tendon pathologies, including Achilles rupture, tendinopathy, and insertional disorders [1-3]. Because several surgical treatments, including V-Y advancement, turn-down flaps, and other tendon transfers, can be used to address Achilles tendinopathy, the prevalence of isolated FHL transfer procedures is difficult to assess [1,3]. Additionally, FHL tendon transfer is not always documented consistently, especially when it is used as an augmentation [4,5]. Regardless, it continues to be a common and effective treatment due to its promising functional outcomes and the adoption of minimally invasive endoscopic techniques [6-8]. 

FHL tendon transfers have been proven to exhibit high levels of overall patient satisfaction, with patient satisfaction rates between 86% and 95% [2,9,10]. Current research focuses on changes observed preoperatively versus postoperatively or compared to a healthy baseline. Several papers have found improvements in patient-reported outcomes following operative management [10,11]. In the past, proven legacy patient-reported outcome measures such as the Foot and Ankle Ability Measure (FAAM) were widely used to assess function and disability in individuals with foot and ankle disorders, including Achilles tendon pathology [12-14]. Patient-reported outcome measures have become increasingly popular, with the Patient-Reported Outcome Measurement Information System (PROMIS) serving as the next generation, as it enhances accuracy and reduces survey burden for patients [15,16]. Studies have been conducted on the use of PROMIS in the measurement of success following orthopedic procedures [2,17,18]. One component of PROMIS is the PROMIS Mental Health (MH) survey. PROMIS MH is used to create a generalized score for a patient’s mental state through a series of questionnaires [19]. Past research has proven mental health and mental well-being to have a positive correlation with clinical outcomes following orthopedic surgeries [20].

Resilience is believed to be an intrinsic, static trait that should not change with injury or surgery. As such, it may provide a strong predictive factor for a patient’s ability to recover postoperatively [21-23]. The current gold standard test for measuring patient resilience is the Brief Resilience Scale (BRS) [1,22,23]. Additionally, prior studies have shown that resilience plays a key role in individuals suffering from chronic musculoskeletal pain and that these patients often have impaired mental health, which may affect the rehabilitation process [24,25]. It is important to note that BRS and PROMIS MH share overlapping psychological domains. However, BRS is specifically designed to capture resilience, whereas PROMIS MH assesses broader aspects of emotional health. While conceptual overlap exists, we believe resilience can contribute unique explanatory value beyond general mental health, which may have clinical relevance in recovery following FHL tendon transfer.

There has been a significant interest in utilizing resilience as a means of predicting postoperative patient outcomes. Several papers have described positive relationships between resilience and patient-reported outcomes following various procedures [2,9-11,17,18]. FHL tendon transfers are technically demanding procedures that are often performed for chronic Achilles tendon pathology in patients who may already have experienced prolonged pain, functional limitations, and failed prior treatments. These conditions make resilience and mental health particularly relevant to postoperative recovery and functional outcomes. To our knowledge, this is the first paper to analyze both preoperative and postoperative resilience following FHL tendon transfer, to directly describe patient resilience postoperatively, and to correlate resilience and PROMIS MH with validated clinical outcomes measured by the FAAM instrument. This paper aims to expand the knowledge surrounding resilience by proving the already theorized characteristics of this trait and to analyze the relationship between BRS and PROMIS MH scores.

## Materials and methods

The research was conducted at the University of Alabama at Birmingham in Birmingham, AL. Following approval by the University of Alabama at Birmingham Institutional Review Board (IRB-300000382), the electronic medical record was searched using the Current Procedural Terminology codes 27680 and 27691 (FHL transfer) for two providers at a single institution between June 2021 and February 2024. Data for all patients were collected prospectively through third-party software and analyzed retrospectively. The initial patient pool comprised 264 patients. Inclusion criteria limited the final cohort to patients who underwent an FHL transfer with a completed preoperative BRS. Patients were excluded if they did not complete baseline PROMIS MH or did not complete at least one postoperative BRS. The final patient cohort included 166 patients. Demographics, including age, sex, and race, were collected. Other races included Asian individuals, or if the patients selected more than one race. The primary outcomes, resilience and mental health, were assessed using validated instruments that have been previously reported among orthopedic surgical populations [26]. Measured outcomes were collected through questionnaires sent preoperatively as well as at two weeks, six weeks, three months, six months, and one year postoperatively. Resilience was measured with the BRS, a series of six questions scored on a five-point Likert scale [22]. PROMIS MH was obtained using the PROMIS version 1.1 computer adaptive test [27,28]. PROMIS MH has a mean T-score of 50 and a standard deviation of 10, representing the general population. A higher score indicates better mental health for this domain of PROMIS. FAAM was obtained through a 21-question subscale assessing activities of daily living (FAAM-ADL) and an eight-question sports subscale (FAAM-Sports), with each question scored on a four-point scale [12,29,30]. A higher number equates to a higher level of physical capacity. 

Statistical analysis

All analyses were conducted by a statistician. Data were collected and organized in Microsoft Excel (Microsoft Corporation, Redmond, Washington, United States) and analyzed using SAS® software (version 9.4, Cary, NC, USA). Descriptive statistics were utilized to assess patient demographics and BRS, PROMIS MH, FAAM-ADL, and FAAM-Sports. A correlation model was used to determine the relationship between resilience, PROMIS MH, FAAM-ADL, and FAAM-Sports scores. A p-value < 0.05 was considered significant. An ad hoc power analysis was performed to calculate a sufficient sample size for statistical analysis. A power analysis was conducted for the difference in mean BRS at baseline (3.68) and one-year follow-up (3.76). The power for the difference was 0.15 based on an N of 50 patients. Power analysis for the correlation between BRS and PROMIS MH for the strongest correlation at (|r|=0.76) is >0.99.

## Results

Following the application of defined study criteria, the final patient cohort included 166 patients. There was an even distribution of patients below and above 50 years of age, with 72.9% female, 41.0% Black individuals, and 54.8% White individuals (Table 1).

**Table 1:** Patient demographics This table documents demographic variables of all patients included within the final patient cohort.

Patient Demographics	Frequency	Percent
Sex		
Female	121	72.9
Male	45	27.1
Age		
<50	80	48.2
≥50	86	51.8
Race and Ethnicity		
Black	68	41
White	91	54.8
Other Race	7	4.2

Reported measurements

**Figure 1:**
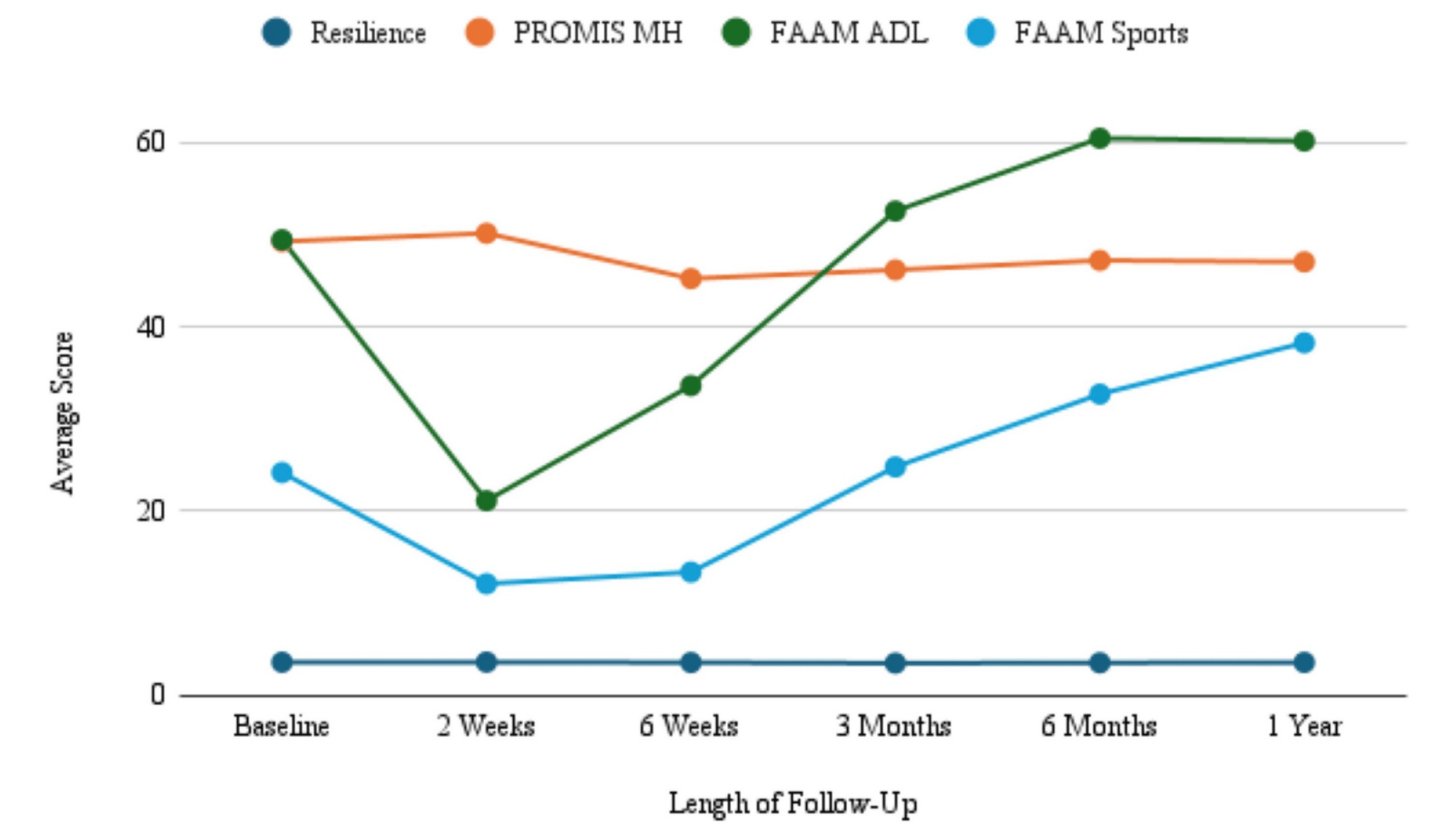
Mean resilience, mental health, and physical function scores during the first year postoperatively The line graph demonstrates overall trends in mean changes across PROMIS MH, FAAM-ADL, FAAM-Sports, and resilience over the defined postoperative intervals up to one year from surgery. PROMIS MH: Patient-Reported Outcomes Measurement Information System Mental Health; ADL: activities of daily living; FAAM: Foot and Ankle Ability Measure

Mean preoperative resilience was 3.65 (0.8046), mean PROMIS MH was 49.22 (9.37), mean FAAM-ADL was 49.41 (21.27), and FAAM-Sports was 24.20 (21.87). The interval changes in resilience, PROMIS MH, FAAM-ADL, and FAAM-Sports are presented in Table 2 and Figure 1. At the two-week follow-up, the mean resilience showed an increase to 3.66 (p=0.6692). Resilience declined from the two-week point to 3.62 at six weeks and 3.55 at the three-month follow-up point (p=0.4431) until it increased at six months to 3.59 (p=0.4349) and at one year to 3.63 (p=0.6624). PROMIS MH increased at the postoperative two-week interval from 49.22 to 50.11 (p=0.9061). There was a statistically significant decrease in PROMIS MH to 45.20 at six weeks (p=0.0001), 46.14 at three months (p=0.0022), and 47.17 at six months (p=0.0188) relative to baseline. At the two-week follow-up, FAAM-ADL and FAAM-Sports both showed significant decreases to 21.14 (p < 0.001) and 12.15 (p < 0.001), respectively. FAAM-ADL increased to 33.61 (p < 0.001) at six weeks, 52.52 at three months (p=0.323), 60.40 (p < 0.001) at six months, and 60.10 (p=0.0014) at one year. Similarly, FAAM-Sports increased to 13.41 (p < 0.001) at six weeks, 24.81 (p=0.853) at three months, 32.69 (p=0.0143) at six months, and 38.25 (p=0.0002) at one year. Resilience was found to be significantly correlated with PROMIS MH at all time points (p < 0.05). The strongest correlation between the BRS and PROMIS MH was seen at one-year follow-up (|r|=.76, p < 0.0001).

**Table 2:** Interval resilience and PROMIS mental health and physical function *- statistically significant change in mean score from baseline using linear mixed-effects modeling (e.g., type III F-tests, t-tests for least squares means, and ANOVA) with p < 0.05 as the threshold for significance. PROMIS MH: Patient-Reported Outcomes Measurement Information System Mental Health; ADL: activities of daily living; FAAM: Foot and Ankle Ability Measure

Patient-Reported Outcome Measure (PROM)	Baseline	2 Weeks	6 Weeks	3 Months	6 Months	1 Year
Resilience	3.65 (0.80)	3.66 (0.73)	3.62 (0.91)	3.55 (0.90)	3.59 (0.86)	3.63 (0.84)
PROMIS MH	49.22 (9.37)	50.11 (8.885)	45.20* (9.36)	46.14* (9.96)	47.17* (9.93)	47.01 (10.36)
FAAM-ADL	49.41 (21.27)	21.14* (20.65)	33.61* (27.18)	52.52 (24.42)	60.40* (24.49)	60.10* (26.07)
FAAM-Sports	24.20 (21.87)	12.15* (22.93)	13.41* (23.40)	24.81 (25.37)	32.69* (27.55)	38.25* (29.42)

## Discussion

Resilience and its relationship to postoperative recovery have garnered significant attention in recent research. However, most existing studies are based on the theory that resilience is unchanging. In this study, we aimed to assess variations in resilience in patients who had undergone FHL tendon transfer surgery and to analyze the relationship between resilience and mental health relative to postoperative changes in patient-reported physical function. Earlier research from our institution has found increased patient resilience to be a positive predictor of outcomes following foot and ankle surgery [21,31]. These papers were limited, as they only utilized resilience measures from a single time point, and postoperative outcomes did not include a reference to mental health scores. The present study identifies a correlation between patient resilience and mental health while demonstrating no significant change in resilience between the follow-up periods. Additionally, the results of this study demonstrate that there was no clear relationship between postoperative mental health and improvements in physical function. Overall, physical function, assessed via FAAM-ADL and FAAM-Sports, improved beyond average baseline scores. Mental health decreased relative to baseline scores and remained relatively consistent through one year.

FHL tendon transfer is a widely accepted treatment for Achilles pathologies [2,3,32-34]. This procedure has been proven to provide strong clinical improvement. De Cesar Netto et al. found significant improvement in patient-reported pain scores and in functional outcomes when compared to the pre-operative state [32]. In addition, one paper found that in patients suffering from a neglected Achilles tendon rupture, treatment with an FHL transfer led to improved sports activity [35].

Historically, research on FHL transfers has used pain and physical function as primary endpoints [3,32,33]. The current study contributes to psychosocial factors that may affect the aforementioned outcomes. It has been previously recognized that knowledge of a patient’s mental attributes can aid physicians in determining which patients are less likely to experience significant improvement following surgery [36]. This review did not include papers on foot and ankle surgery or those analyzing resilience. We are adding to the knowledge of the effect of mental health on surgical outcomes by introducing another measured outcome in resilience and by correlating it with mental well-being. Although no standardized guidelines exist to address emotional and social challenges, the identification of patients with more adverse psychosocial factors, including decreased resilience, may enable more accurate predictions of postoperative outcomes and influence management strategies.

The first study to describe the effect of resilience on orthopedic surgeries found a strong correlation between increased resilience and better patient-reported outcomes [37]. Our study utilized both preoperative and postoperative BRS scores, allowing us to analyze trends from the preoperative to postoperative state. Several papers have been conducted on the use of resilience in predicting operative outcomes in orthopedics [18,21,38,39]. These studies have consistently identified a direct, positive correlation between resilience and patient-reported outcomes, indicating that high preoperative resilience is predictive of greater improvement in physical function and pain after surgery [38-40]. These past studies were all conducted using only a single resilience value taken either preoperatively or postoperatively [38-40]. While validation studies of the BRS show good reliability and construct validity, there is little evidence of its responsiveness to change in detecting small longitudinal shifts within individuals. With the limited response range and item count of the BRS, subtle incremental changes may not register as score differences if the change does not push responses across categorical thresholds. Nevertheless, our study adds to recent literature by investigating whether resilience is affected by surgical experience. Our findings showed no significant changes in resilience, reinforcing the validity of using a single resilience score, regardless of timing, as a predictive factor for surgical outcomes. 

Mental health and well-being have been established as predictive factors for surgical outcomes in orthopedics [36]. In our study, we utilized PROMIS MH surveys to assess the general mental health status of each patient. Although PROMIS has been validated through the National Institute of Health as a superior test for recording orthopedic patient outcomes, prior literature has not been able to establish a minimal clinically important difference (MCID) for PROMIS MH to determine whether statistically significant differences are clinically significant [32]. Nonetheless, several studies have been conducted on the association between patient mental health and postoperative outcomes [18,20,36,41]. One study by Hines et al. investigating the relationship between mental well-being identified a correlation between higher preoperative Veterans RAND 12-Item Health Survey Mental Component Scores and the likelihood of experiencing significant clinical benefit [20]. Resilience most likely contributes to the overall mental well-being of a patient and adds to their predictive capabilities.

This paper has several strengths and limitations. This study is subject to response biases, including attrition bias, since measured outcomes were collected via questionnaires sent through text and/or email. It was not within the scope of this study to assess the relationship between patient factors, such as comorbidities, and changes in patient resiliency. Therefore, our statistical analysis did not stratify patients according to these factors. As such, we are unable to report how these secondary factors may influence changes in resilience. Given our defined inclusion and exclusion criteria, the size of the cohort was limited after isolating for FHL transfer surgery. These criteria were necessary to isolate resilience from potential confounding factors, including surgical site or type. However, the analysis performed is supported by an ad hoc power analysis to confirm that an adequate patient population was obtained.

## Conclusions

Resilience has a significant relationship with a patient's mental health and shows no changes between the preoperative and postoperative states following FHL transfer. As one of the first studies to verify the stability of this trait and to correlate it with mental health, this paper contributes valuable validity to prior and future studies using preoperative resilience as a means of predicting postoperative outcomes following foot and ankle surgery.
